# Boosting Creativity through Users’ Avatars and Contexts in Virtual Environments—A Systematic Review of Recent Research

**DOI:** 10.3390/jintelligence11070144

**Published:** 2023-07-17

**Authors:** Jiayin Liu, Jean-Marie Burkhardt, Todd Lubart

**Affiliations:** 1 LaPEA, Université Paris Cité and Univ Gustave Eiffel, 92100 Boulogne-Billancourt, France; jiayin.liu@etu.u-paris.fr; 2 LaPEA, Univ Gustave Eiffel and Université Paris Cité, 78008 Versailles, France; jean-marie.burkhardt@univ-eiffel.fr

**Keywords:** virtual environment, creativity, avatar, virtual context, embodiment, presence

## Abstract

As an artificial space extended from the physical environment, the virtual environment (VE) provides more possibilities for humans to work and be entertained with less physical restrictions. Benefiting from anonymity, one of the important features of VEs, users are able to receive visual stimuli that might differ from the physical environment through digital representations presented in VEs. Avatars and contextual cues in VEs can be considered as digital representations of users and contexts. In this article, we analyzed 21 articles that examined the creativity-boosting effects of different digital user and contextual representations. We summarized the main effects induced by these two digital representations, notably the effect induced by the self-similar avatar, Proteus effect, avatar with Social Identity Cues, priming effect induced by contextual representation, and embodied metaphorical effect. In addition, we examined the influence of immersion on creativity by comparing non-immersive and immersive VEs (i.e., desktop VE and headset VE, respectively). Last, we discussed the roles of embodiment and presence in the creativity in VEs, which were overlooked in the past research.

## 1. Introduction: The Rise of VR in Creativity Research

With the evolution of human-beings and the development of human society, the impact of humans’ activities on nature is increasingly obvious. Successive major innovations since the 20th century(e.g., Individual vehicles, Computers, Internet, Autonomous Systems, etc.) have changed not only the way we live but also impacted the physical world. Information technology is one of the main trends driving the Third Industrial Revolution. Following academic research that developed the first head-mounted displays and computer graphical interfaces (Sutherland 1964, 1965), the invention of video games such as Pong and Maze War in the early 1970s symbolized the introduction of virtual worlds into the social world. Recent years have seen the emergence of hybrid systems involving virtual, augmented or mixed reality to create increasingly immersive user experiences. Thus, the Anthropocene period--the “age of Humans” as a central force in planetary change--is characterized by the creation of new virtual workspaces and leisure spaces, shaping the social space and organizations, in addition to human impact on the physical world.

Virtual Reality (VR) systems are high-end user-computer interfaces that support real-time stereoscopic environmental simulation and user-to-user or user-to-object interactions through multi-sensory channels (Burdea and Coiffet 2003), though most Virtual Environments (VEs) restrict visual and auditory simulation (Melo et al. 2022). The VE is more or less superposed with the physical environment and offers a wide range of possibilities to support humans’ activities by possibly changing physical (e.g., distance, gravity, size) and/or social constraints (e.g., appearance, anonymity). VEs serve as new spaces for human-beings to reshape the relationship with the physical environment as well as explore innovative solutions to existing environmental problems in the age of Anthropocene.

Some VEs are designed to support one single user whereas Multi-User VEs (MUVEs) support interactions among several users connected through them. VEs can be classified based on the type of devices and interactions that range from desktop applications with conventional computers, to Head-Mounted-Displays (HMDs) with trackers or Cave Automatic Virtual Environment (CAVE) configurations where the users’ whole body may be engaged in interactions with the digital representations of objects and the environment relevant for the task at hand. Each specific technological configuration of devices is associated with a specific level of immersion^1^, which refers to a subset of sensorial inputs and feedbacks controlled by the interface of the system, and their quality and consistency (Burkhardt 2003; Slater 2003; Stoffregen et al. 2003), given that it has been claimed that “The more that a system delivers displays (in all sensory modalities) and tracking that preserves fidelity in relation to their equivalent real-world sensory modalities, the more that it is ‘immersive’.” (Slater 2003, p. 1).

Another important feature of VE is the digital representation of users and contextual elements that may be represented and behave similarly—more or less, or even not at all—to what can be expected in the physical environment. The interaction with objects, elements and other users presented in the VE is most of the time supported with users’ digital representations (i.e., avatar).

Creativity, as an essential skill for the 21st century (Happ 2013; Puccio 2017; Nakano and Wechsler 2018), promotes the productivity and efficiency at both individual and collective levels (Sternberg and Lubart 1999) in numerous domains such as the domains of education and industry (e.g., Mumford and Simonton 1997; Fasko 2001; Moran 2010; Zhou and Hoever 2014). VEs present materials and resources that can support creativity in at least three ways (Burkhardt and Lubart 2010): (a) to help people develop skills related to creativity or creative thinking; (b) to engage people in new kinds of experiences; and (c) to support people’s creative process and performance while engaging in a creative task, which is the main focus in this review.

Several literature reviews (Bourgeois-Bougrine et al. 2022; Gong et al. 2022) have claimed that VR systems enhance creative performance for individuals or groups by providing more possibilities with their specific features (e.g., anonymity, tracing, digital representations) and immersion. However, the effect of presence and embodiment, two central concepts describing the subjective user experience in VEs, have not been examined related to creativity. 

This literature review aims to identify and summarize existing effects and mechanisms involved in enhancing the creative process and performance related to digital representations (i.e., user-representation and contextual-representation), embodiment and presence in VEs. These include the Proteus effect, Self-Similarity, Social Identity Cues, the Simulation effect of physical environment, Priming and embodied^2^ metaphor. Furthermore, we summarize the difference between creative performance in immersive and non-immersive VEs to provide empirical results on the influence of immersion on creative activities, and examine the role of embodiment and presence, two psychological states impacted by immersion, in the virtual creative ecological system,

The paper is organized as follows: First, we introduce the main concept related to research on creativity and VEs, with a focus on the latter’s features (i.e., non-immersive and immersive VEs, digital representations, presence, embodiment). Second, we describe the review method based on PRISMA guidelines (Page et al. 2021). Third, we report and discuss empirical results of the selected studies related to immersion, user representation, embodiment, contextual representation, and presence. Finally, a conclusion based on the results summarized in this paper, and limitations of current studies as well as a forward-looking perspective on the orientation of future studies are proposed.

## 2. Research on Creativity in VEs

### 2.1. Creativity, Creative Processes and Measures

**Figure 1:**
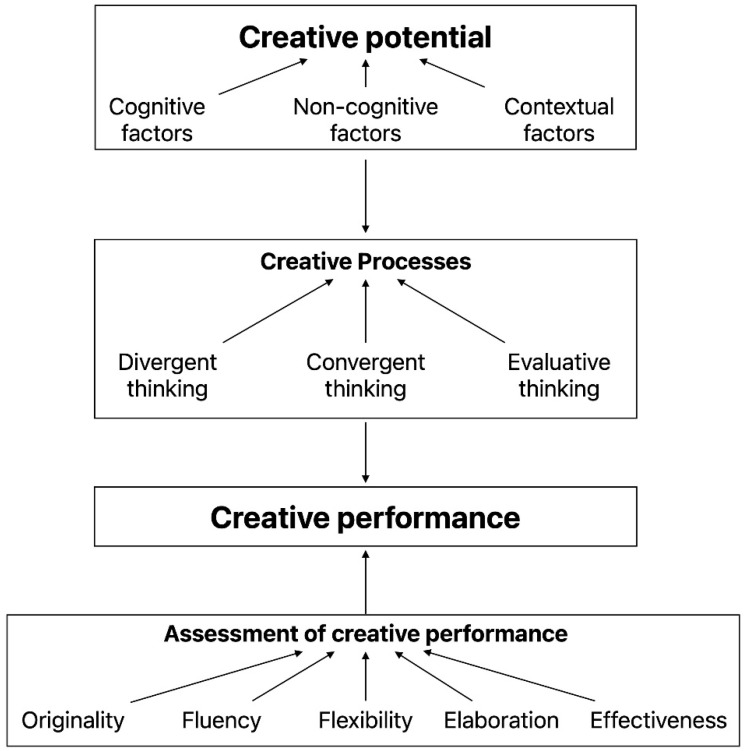
An adapted flow chart of the multivariate approach to creativity (Adapted from Figure 15.1 of Lubart and Thornhill-Miller 2019).

Creativity has been defined as “the ability to produce work that is both novel (i.e., original, unexpected) and appropriate (i.e., useful, adapted to task constraints)” (Sternberg and Lubart 1999) (summarized in Table 1 with other definitions mentioned in this section). According to the 7Cs approach (Lubart 2017), creative performance results from a creative process and creative potential (cf. Figure 1). The creative process is defined as the sequence of thoughts and actions that lead to a creative production (Lubart 2001). The creative process varies depending on the individuals involved in the creative production and the given creative tasks (Lubart 2017).

Guilford (1956) emphasized the importance of three creative processes (i.e., divergent thinking, convergent thinking, and evaluative thinking) in the Structure of Intellect (SOI) theory, which influenced the assessment of creative performance. Divergent thinking refers to a process that people engage to generate ideas in multiple domains seeking a large number of ideas. It provides the target of numerous classical assessments of individual creativity such as *The Torrance Tests of Creative Thinking* (TTCT) (Torrance 1998) and *The Alternative Uses Test* (AUT) (Guilford 1956). In contrast, convergent thinking refers to a process in which people seek a unique answer or conclusion in a given context. A classical assessment of convergent creative thinking is the *Remote Association Test* (RAT) (Mednick and Mednick 1971), in which participants are given three words and are asked to give a fourth word that is associated with the three given words. In addition, evaluative thinking refers to an evaluation of the results of creative thinking in terms of ideas’ effectiveness, goodness, or suitability. Most creative tasks require various combinations of divergent and convergent thinking processes as in creative problem-solving techniques, brainstorming sessions and design tasks. For example, the stages of the design process are associated with both divergent thinking (e.g., Prototype generation stage) and convergent thinking (e.g., Evaluating outcome stage, Final outcome phase) (Lazar 2018).

The assessment of creative performance is usually conducted using the Consensual Assessment Technique (CAT) (Amabile 1982) with one or several of the following categories: (1) Originality, which refers to the infrequency of the idea, often judged by either experts in the relevant domain or calculated with specific metrics; (2) Fluency, which refers to the total number of ideas generated in the given time lapse, often calculated with specific metrics; (3) Flexibility, which refers to the number of categories of generated ideas (e.g., furniture, tools, etc.), often calculated with specific metrics; (4) Elaboration, which refers to the extent that details are given by the participant for each idea, often calculated with specific metrics; and (5) Relevance, which refers to the extent to which ideas fit the constraints of the given task and reflect classical facts and principles (Cropley and Cropley 2008; Guilford 1956).

Environment-centered factors are considered as one of the three crucial categories of ingredients that impact creativity in the model of multivariate approach to creativity (Lubart 2017). The characteristics such as light, colors, windows, and plants influence employees’ self-reported creative potential (Ceylan et al. 2008). A study examining the relationship between the creativity-supporting working environment and innovative performance with a sample of 103 firms found that the creativity-supporting working environment in which the physical creative elements (e.g., furniture, daylight, window view, etc.) exist is associated with greater innovation (Dul and Ceylan 2014). In addition, a view on nature has a positive impact on creativity. In Chulvi et al. (2020), students produced more ideas in real and simulated natural environments than in a conventional classroom. Beyond the environmental features, social-contextual features also influence creativity. For example, a high level of autonomy enhances intrinsic motivation which is an important ingredient for creative potential (Gagné and Deci 2005; Lubart 2017). Furthermore, aversive leadership and an unsupportive organizational climate are negatively related to creative performance in workplaces (Choi et al. 2009).

Although this multivariate model is compatible with both individual and collective creativity, there are differences between individual and collective creativities. Collective creativity is defined as “the process through which two or more people, often with different or complementary skills, engage in shared creation, frequently producing something that they could not or would not produce on their own.” (Lubart and Thornhill-Miller 2019, p. 286). The group creative output is not simply a sum of individual creative outputs. Instead, benefiting from shared knowledge and support from each other, a collective creative activity has a “1 + 1 > 2” effect compared to individual creative activities (Parjanen 2012). The collective creative process is influenced by two factors: (1) group size: the smaller the size of the group, the more creative the group is; (2) homogeneity of group members: the more diverse the expertise of group members, the more creative the group is (Richard 2016).

### 2.2. VR Systems: New Tools to Enhance Creative Performance

Recent literature reviews (Akdaş and Çalgüner 2021; Bourgeois-Bougrine et al. 2022; Gong et al. 2022) suggest that VEs provide potentially new ways to enhance creativity. Akdaş and Çalgüner summarized the application of VR technology (i.e., desktop, HMD, CAVE) in product design (Berni and Borgianni 2020) in research between 1997 and 2021, showing the dominance of HMD over desktop VR and CAVE. In addition, VR technology was more used for virtual assembly and prototyping, mechanical simulation, and production evaluation than other design purposes. However, the effectiveness of the VR technology in product design was not assessed. Based on a systematic review of the affordances of virtual brainstorming from the perspective of creativity, Gong et al. (2022) identified the following eight affordances of VE that help support brainstorming sessions: anonymity, appraisal, avatars, immersion, multiple communication methods, recording, simulated objects, and tracing. The article did not examine creative performance in certain papers, which made it difficult to make a conclusion about how effective the affordances are in helping to enhance creative performance. Bourgeois-Bourgrine and colleagues analyzed research based in Europe between 2014 to 2021 studying the effect of immersive VE on individual and collective creativity. The results suggested that avatars and contents in VEs are useful features to enhance creativity. In conclusion, VR platforms are effective tools to support different types of creative activities (i.e., product design, brainstorming, individual creative production). The paper provided evidence through empirical studies showing that VR technology enhances creative potential (e.g., motivation, knowledge to the creative task) and creative performance (e.g., relevance, originality, elaboration).

### 2.3. The Effect of Digital Representation, Embodiment and Presence on Various Dimensions of Creativity in VEs

Because users of VR platforms are usually distant, they need to have a digital representation in the virtual environment so that they can locate themselves and interact with objects and other users. Existing digital representations of users can be classified into three main types (Kadri et al. 2007): (1) a tool (e.g., a pen); (2) a whole or subpart of the body (e.g., humanoid); and (3) an object without semantic content (e.g., a cursor). VR has thus an important feature of enabling anonymity and/or manipulating information about users’ identities. Indeed, users can choose or not to display some of their personal information (e.g., name, appearance), which makes them less identifiable if they choose not to do so (Jaidka et al. 2022). Anonymity is one of the conditions that cause deindividuation (Zimbardo 1969) which can be defined as “a psychological state in which people lose their self-consciousness” (Singer et al. 1965, p. 356). Alternately, users digital representations can contain identity cues that are the same or differ from reality. 

At the individual level, VEs provide a context in which users can re-identify themselves with new identities through avatars (Yee and Bailenson 2007), which might induce specific identification effects (e.g., Proteus effect) and enhance their creative performance.

Avatar Identification refers to an emotional and cognitive attachment between the user and his or her digital representation (Cohen 2001). It requires the users to mask their own identification and embody themselves through avatar identification. Avatar identification has two aspects: perceived similarity and wishful identification (Feilitzen and Linné 1975; Van Looy et al. 2010).

Perceived Similarity refers to the fact that people tend to identify with avatars through salient common characteristics such as looks, clothing, or a social situation. The results of a survey examining the relationship between user avatar differences and avatar identification with 666 respondents showed that player-avatar similarity is positively related to avatar identification, and avatar identification is positively related to game enjoyment (Trepte and Reinecke 2010). As positive affect is one of the components that reflect the level of game enjoyment (Gajadhar et al. 2010) and results in improved performance in creative problem-solving tasks (Isen et al. 1987), it might be able to explain the reason why using self-similar avatars enhances creative performance. That is, a self-similar avatar increases positive affect by enhancing avatar identification, which leads to better creative performance. However, there is still little empirical evidence that supports this mechanism.

Another identification effect at the individual level is the Proteus effect. The Proteus effect is defined as the phenomenon in which one’s behavior conforms to the perception of external expectations of the avatar (Yee and Bailenson 2007). For example, a user with an attractive avatar walked closer to the confederate and disclosed more personal information than a user with an unattractive avatar in the VE (Yee and Bailenson 2007, Experiment 1). The Proteus effect occurs in an anonymous environment (e.g., VE) where the individual identity of users is filtered. In this case, deindividuated users conform their behaviors to the social norm which can be recognized based on the characteristics of their digital identification (Yee and Bailenson 2007). However, the Proteus effect is restricted when users embody avatars with undesirable characteristics, that is, the users only adapt to the desirable identity cues from their avatars (McCain et al. 2018). It might be explained by wishful identification (Praetorius and Görlich 2020), which refers to a desire of users to reflect the ideal self in the avatar and choose the avatar with characteristics they want (e.g., attractiveness) (Van Looy et al. 2010; Takano and Taka 2022). The effect can be explained with the Self-discrepancy theory introduced by Higgins, in which a salient gap between actual self and ideal self induces negative emotions, and the enjoyment of identification is based on the reduction of the gap (Higgins 1987). Therefore, choosing an avatar with a desirable identity reduces negative emotions and increases enjoyment with the chosen avatar.

At the collective level, the use of digital representations (i.e., avatars) that share the same social identities in a group lead users to re-identify themselves as part of the group with given social identity cues rather than individual identity cues which are applied with the theory of avatar identification, which shares the same mechanism as the social identity theory (Tajfel et al. 1979) when solving creative problems together. According to this theory, people categorize social groups depending on the emotional involvement and shared characteristics among group members. Identifying oneself as an in-group member helps a person to increase the motivation to serve the group, collaborate with other group members and contribute to the given task (e.g., creative task) (Guegan et al. 2017a). 

Context and content digitally represented in VR also influence creative performance in two ways: (1) by arousing cognitive functions (e.g., attention) that are related to creativity such as presenting the view of nature or the visual dynamic environment, and (2) by providing contextual cues that are linked with creative thinking such as the priming effect and embodied metaphor.

Numerous studies have shown the effectiveness of the view of nature on enhancing creativity (e.g., Atchley et al. 2012; Jones 2013; Plambech and Van Den Bosch 2015). Moreover, including natural elements in indoor environments also enhances creative performance (Alawad 2012; Chulvi et al. 2020; e.g., McCoy and Evans 2002). According to Attention Restoration Theory (ART), natural elements help restore directed attention fatigue as it requires a different type of attentional processing compared to the typical attentional demanding environments in people’s daily life (Crossan and Salmoni 2021). The restored direct attention is required for idea generation and evaluation stages when engaging in creative activities. Visual dynamic stimuli in VEs can also be considered as a feature that enhances creativity. This can be explained by cerebellum-related functions. The cerebellum plays an important role in motor control and perception (Paulin 1993) and also has a positive impact on creative activities (Gao et al. 2020; Jalil 2007; Vandervert et al. 2007). Therefore, the presence of moving visual stimuli enhances creative performance by evoking the function of Cerebellum. The two effects can be induced in VEs in which the related stimuli are activated through immersion, as an important feature of VE, leading users to think that they are actually in the VE and behave as they are in the reality.

Beyond evoking cognitive functions to increase creativity, people can also develop VEs that include creativity-related contextual cues and enhance creative performance with Priming effects or embodied metaphor effects.

Priming is described as the secondary activation of knowledge structures by the current context which triggers cognition and behavior (Bargh et al. 1996). In the domain of creativity, primed elements are used to provide examples for creative tasks that follow (Marsh et al. 1999) and enhance the flexibility of creative performance, as well as impacting convergent thinking process because the priming effect activates remote associations (Sassenberg et al. 2017). However, creative performance influenced by the priming effect is restricted as the generated ideas show homogeneity with the provided example in the priming stage (Rubin et al. 1991). In VE, instead of the traditional way (i.e., exposing the priming objects before the task), the priming stimuli can be merged with the general environment (i.e., contextual priming) (Bhagwatwar et al. 2018). This setting allows people to receive information from the stimuli during the whole process of their work instead of only in the pre-task process. 

Moreover embodying metaphor–that is prompting the user to move or act in order to enacting metaphors dedicated to foster creativity such as “think outside the box”-can be used in VR to enhance creative performance. Metaphors are used to support creativity for several reasons: (1) metaphors provide information that leads people to think about the problem from another angle, (2) metaphors induce or extend an original insight to a problem, and (3) metaphors express new ideas in a familiar and acceptable way for a wide range of people (Lubart and Getz 1997). A conventional way to induce an embodied metaphor is to ask people to move or pose in a certain way to enact the metaphor through their body. For example, students were asked to sit inside or outside of a box to present the metaphor “think outside the box” (Leung et al. 2012). A physically embodied metaphor is restricted as it must ensure the safety of the participants. In contrast, in a VE, people can experience “dangerous” metaphors in a safe way. For example, in the experiment designed by Wang and colleagues (Wang et al. 2018), participants were asked to break walls that appeared in front of them, which was hard in real life but feasible in a VE. 

From the technical perspective, digital representations in VEs enhance creative performance in various ways. However, subjective experience of users (e.g., motivation, joyfulness, embodiment, presence) in VEs impact creative performance as well from a cognitive perspective. This review focuses on two subjective experiences, presence and embodiment in VEs which are influenced by the level of immersion of the VR systems.

Embodiment, which is related to the avatar is an important factor that influences motivation, perception, cognitive activities, and creativity-enhancing effects (e.g., the Proteus effect) in VEs (Yee and Bailenson 2009; Steed et al. 2016; Flavián et al. 2021; Keenaghan et al. 2022). Embodiment is defined as a subjective feeling of being in a virtual body and having the property of that body (Kilteni et al. 2012). It can be measured by validated self-report questionnaires (e.g., Roth and Latoschik 2019) and physiological activity recorders (e.g., fNIRS (Wang et al. 2018)). Embodiment involves three dimensions. The first one is the sense of self-location, which refers to one’s spatial experience of being inside of a virtual body. The second one is the sense of agency, a subjective sense of motor control over one’s avatar. The last one is the sense of body ownership, which means a feeling of self-attribution to the virtual body. Different virtual settings (e.g., field of view, movement synchronization) induce different levels of embodiment. The more the three facets are aroused, the more an embodied experience is induced (Kilteni et al. 2012). A higher level of embodiment might lead to a greater Proteus effect (Yee and Bailenson 2009) for individuals and the effect of social identity cues in teamwork, as well as an indirect enhancement of creative productivity.

Being embodied in a different virtual body from the user’s actual body changes his or her cognition. For example, white people embodied in a black body showed stronger empathy and lower race bias to black people (Peck et al. 2013; Banakou et al. 2016; Thériault et al. 2021). Similarly, being embodied in an elder avatar induces empathy and reduces implicit bias to older people (Banakou et al. 2018). Embodiment changes also behaviors such as cognitive performance (Banakou et al. 2018) and walking speed (Reinhard et al. 2020). Embodiment is an essential factor and an effective predictor of the Proteus effect. Yee and Bailenson (2009) designed an experiment to examine the importance of embodiment in the Proteus effect. Participants were assigned to either mirror or playback conditions. The only difference between the two conditions was that in the mirror condition, participants saw the avatar in the mirror with synchronized movement for what they did, whereas in the playback condition, the movements were asynchronous (i.e., what participants saw was the playback of movements of the last participant). The results showed that a synchronized digital avatar (i.e., embodiment condition) resulted in a greater change of behavior than “being in” an asynchronized virtual body (i.e., non-embodiment condition). 

In contrast with embodiment, presence which is more associated with a virtual context is an important indicator to evaluate the user experience in a VE (North and North 2016). Presence refers to “a state of consciousness, the [psychological] sense of being in the virtual environment” (Barfield et al. 1995). It can also be measured with questionnaires (Makransky et al. 2017) and physiological activity recorders (e.g., EEG (Clemente et al. 2014)). Presence can also be divided into three types: spatial presence, social presence, and co-presence (Bulu 2012). Compared to nIVEs, users pay full attention to stimuli in IVEs, which results in greater spatial presence (Bystrom et al. 1999). High attention and presence can positively influence the creative outcome (Heldal et al. 2007). At the collective level, social presence and co-presence also play an important role in user experience and performance. Social presence can be described as the extent of the salience of other persons and the interpersonal relationship in the interaction (Short et al. 1976), whereas co-presence is defined as the perception of being in the same virtual environment with others (Goffman 2008). Different from the sense of presence, social presence and co-presence emphasize the psychological connection among users (Nowak 2001). In a MUVE, spatial presence, social presence, and co-presence are positively correlated with each other (Bulu 2012).

In the nIVE, users experience a narrow presence in the VE without totally excluding the real environment (Okeil 2010). In contrast, with an IVE, users perceive the stimulus through their natural sensorimotor system and are isolated from the real world (North and North 2016; Han et al. 2022). However, the mechanism of the impact of presence on creative performance in the VE is not clear. 

**Table 1:** Definitions of main creativity- or VE-related concepts of the article.

Factors	Definitions
Creativity	the ability to produce work that is both novel (i.e., original, unexpected) and appropriate (i.e., useful, adaptive concerning task constraints)
Creative process	the sequence of thoughts and actions that result in a creative production
Collective creativity	the process through which two or more people, often with different or complementary skills, engage in shared creation, frequently producing something that they could not or would not produce on their own
Deidentification	a psychological state in which people lose their self-consciousness
Proteus effect	a phenomenon that one’s behavior conforms to the perception of external expectations to the avatar
Embodiment	a subjective feeling of being in a virtual body and having the property of that body
Presence	a state of consciousness, the [psychological] sense of being in the virtual environment
Immersion	A subset of sensorial inputs and feedbacks controlled by the interface of the system, their quality and consistency

## 3. Method

The literature review analyzes how digital representations (i.e., user-representation and contextual-representation) affect creative thinking and performance in non-immersive and immersive VEs. Moreover, we discuss the role of embodiment and presence in the virtual creative environment.

As the tools which support individual and collective creativity in a VE are developing rapidly, we decided to only analyze studies in the past ten years (2013–2022). We searched for articles in two major databases, PsycINFO and Google Scholar. We used the following combinations of keywords: (“creativity” OR “creative”) AND ((“virtual reality” OR “virtual environment”) OR (“virtual” (“immersive” OR “immersion” OR “presence”)) OR “embodiment”). In addition, we searched articles that were mentioned in the previous literature reviews related to the creativity and VE (e.g., Gong and Georgiev 2020; Bourgeois-Bougrine et al. 2022). We obtained 483 articles after removing the duplicates in the identification process. 

**Figure 2:**
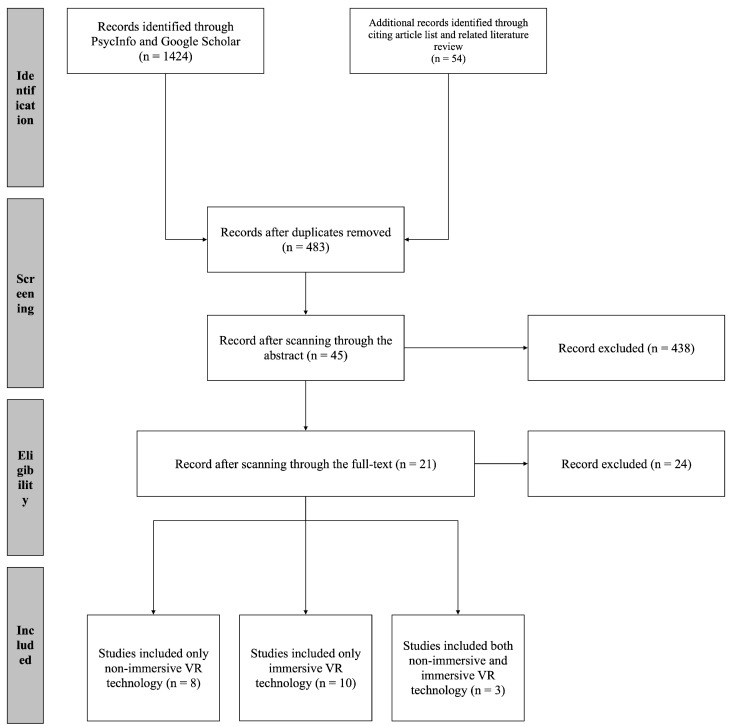
The PRISMA flow chart for the literature selection processes.

The screening process was organized in two steps (cf. Figure 2). First, we screened the articles by reading the topics and abstracts and excluded 438 articles whose research topics did not cover VEs and creativity. Then we selected the articles with full text referring to the guidelines of the PRISMA statement. The selected paper had to match all the *inclusion criteria:* (1) The apparatus of the study must contain a desktop and(or) an HMD device; (2) The research must include one or several of the following topics: contextual-representations, user-representations, immersion, embodiment, presence; (3) The study must study creative activities; (4) The study must include a sample of more than 30 participants; (5) The study must be published in English. Furthermore, the studies must not match any of the *exclusion criteria*: (1) The experimental method is not clear (e.g., the apparatus not specified); (2) The study did not report clearly data (e.g., sample size, gender, age, etc.). 24 articles were excluded for the following reasons: (a) the studies were not related to target research topics (*n* = 6); (b) the studies included a small sample size (*n* = 13); (c) the article was repeated (*n* = 1); (d) the methods of the studies were not clear (*n* = 4). In total, 21 articles were selected for the analysis in this review. Almost half of the studies use only an immersive technology VR (*n* = 10), while the next most represented studies use only non-immersive VR technology (*n* = 8). Only 3 studies use both non-immersive and immersive VR technology.

## 4. Analysis

Firstly, we describe the core publication characteristics, in particular in relation with the VR technology involved in the studies. Second, we examined the articles content in order to address the following main 3 topics: (1) difference(s) of creative performance in non-immersive and immersive VEs; (2) influence of digital user representation, i.e. avatar on creativity; (3) influence of virtual contextual cues on creativity.

For the latter aspects of digital representations, three subtopics are discussed: (a) the general use of digital representations in increasing creativity, (b) the application of creativity-boosting theories in the VEs (e.g., the Proteus effect and embodied metaphor), and (c) the psychological states (i.e., embodiment and presence), which also impact creativity enhancement.

### 4.1. Main Characteristics of the Selected Studies

**Figure 3:**
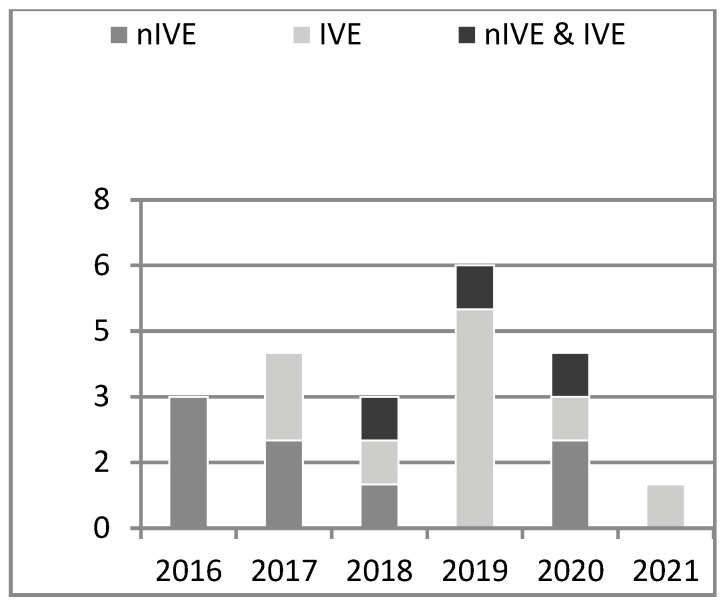
The number of articles using different VEs each year (y axis = number of publications, x-axis = year of publication).

Although the range of publications that we searched covered 2013 to 2022 in the paper selection process, the first article studying the influence of the nIVE on creativity was published in 2016. Researchers started to use the IVE as a tool to enhance creativity in 2017. No study examining the creativity-enhancing effect in only nIVE was found since 2021. Instead, researchers conducted experiments to either examine the effect of the creative IVE, or compare two VEs (i.e., nIVE/IVE) on creativity enhancement (cf. Table 2 and Figure 3). One explanation could be the immersive VR technology became more user-friendly and affordable in recent years, and nIVEs on the computer screen are losing their strength in boosting creativity because of their limitation, such as the inconsistent body scale of the users and their avatars (Hong et al. 2016). Over two-thirds of selected articles in this review are journal articles (*n* = 13). Other types of articles include conference proceedings (*n* = 3), book sections (*n* = 3), and doctoral dissertations (*n* = 2).

In studies related to nIVE, researchers tended to use pre-developed software as the experimental apparatus. Within the desktop VE simulators, Second Life was most used for the studies, whereas Open Wonderland was also applied. In contrast, most studies exploring the effect of IVE used self-developed scene or software, which were designed for the research purposes. Other software, such as Gravity Sketch, and VEnvl, were also used in the IVE-related-studies. Around 3/4 of the studies that used nIVE studied collective creativity, whereas over 4/5 of studies using IVE or the difference between nIVE and IVE focused on individual creativity. The dramatic difference of research interests in two VEs might have resulted from the difficulty to build a stable network system on headsets. Unlike the desktop, there is still little software supporting the VR platform and building a multiplayer VR platform is time-consuming. Around half of immersive-virtual-environment-related studies used HTC Vive as the VR platform, and the second most widely used device in such studies was Oculus Rift. When it comes to the type of creative tasks conducted in the studies, two-thirds of studies used divergent thinking tasks (e.g., cardboard boxes task, Alternative Uses test) and brainstorming sessions to measure individual and collective creative performance, respectively. Further, one third of studies asked participants to engage in a product design activity that required the participants to visualize their ideas instead of simply listing them (e.g., choreography (Parmar 2018) or a bus stop (Hong et al. 2016)) (see Table 3 and Table 4 for the methods used in the studies examining individual and collective creativity, respectively).

### 4.2. Immersive and Non-Immersive VEs: Which Better Enhances Creativity?

We found only three papers that examined differences between IVE and nIVE in terms of their support of creative activities (Obeid and Demirkan 2020; Palanica et al. 2019; i.e., Parmar 2018). Parmar (2018) conducted a series of experiments to compare three VEs (i.e., desktop, headset without avatar, and headset with avatar) in terms of the learning process and creativity. The results showed that students had higher scores in the creative task in IVEs in VR headsets compared to a desktop VE where they were provided with 2 dimensional materials. Similarly, Obeid and Demirkan (2020) conducted a between-subjects experiment to compare the creative potentials (flow, spatial ability, motivation) in nIVE and IVE that present the same contents (i.e., two versions of the Gravity Sketch software). Results showed that the flow, spatial ability, and motivation of participants were higher in IVE than in nIVE, which suggested the dominance of IVE in providing a creativity-supporting environment. However, the results of Palanica et al.’s (2019) experiment did not align with the previous two studies. In this experiment, participants were asked to watch the videos presenting either urban or natural views on either a tablet (i.e., nIVE) or VR headset (IVE). No significant difference between nIVE and IVE was found in terms of their influence on the fluency, flexibility, elaboration and originality of the ideas generated during watching videos. A possible interpretation for the inconsistency among studies comparing IVE and nIVE could be that in Palanica et al. (2019)’s study participants only received passively the visual information provided by nIVE or IVE, whereas in the other two studies (i.e., Obeid and Demirkan 2020; Parmar 2018), participants were asked to interact with the VEs by either dancing with avatars or completing a design task. Therefore, interaction might be an important condition to show the advantages of IVE in enhancing creative performance.

### 4.3. Digital User Representation: The Role of Avatar

#### 4.3.1. The Use of Avatars in VEs

The shape and content of avatars vary across studies, depending on the types and configuration of VEs and the activities in which users engage in a VE (Burkhardt et al. 2005). Under some conditions, the avatar is absent in the VE. For example, in a study comparing natural and urban contexts in nIVE and IVE on creativity (Palanica et al. 2019), participants were asked to sit on chairs and to observe the VE by moving their head instead of navigating or interacting with the environment. In two other experiments examining the influence of contextual cues in VEs on creative performance (Guegan et al. 2017b; Nelson and Guegan 2019), participants were also asked to observe the VEs without interacting with them. In such experiments in which people received passively the environmental information, the avatar might be absent. When the main objective of the task is to design a product or draw a sketch in a VE, the avatar is usually represented by two virtual controllers with tool bars on them (Obeid and Demirkan 2020). 

However, when the main objective of the studies was to have communication, interaction, and collaboration in the VE, avatars were used. Users experience self-representation (Yee and Bailenson 2007) and enhance co-presence (Aseeri and Interrante 2021) through avatars. A virtual brainstorming is a social gathering scenario in a VE. When participants are asked to participate in the brainstorming sessions in a VE, they are provided with either generic avatars that match their gender (Bhagwatwar et al. 2018; Bourgeois-Bougrine et al. 2020) or avatars with more identity cues to induce more specific user-representation and change behavior (Guegan et al. 2016; De Rooij et al. 2017; Guegan et al. 2017a; Marinussen and de Rooij 2019; Buisine and Guegan 2020). 

In summary, the avatar might not be presented when participants passively receive information transferred by the VE. However, when participants need to interact with the VE, an avatar (controller, tool, or humanoid) plays a role to help people monitor the position and the scale of self and objects in the VE (e.g., Hong et al. 2016), or to provide individual and social identity cues to induce creativity-enhancing effects (e.g., Guegan et al. 2016, 2017a; Marinussen and de Rooij 2019).

#### 4.3.2. The Effects induced by Human-like Avatars on Creativity

The results of an experiment conducted by Marinussen and de Rooij (2019) showed that self-similar avatars help increase originality (cf. Table 5). In this study, participants were asked to create either a self-similar or a non-self-similar avatar. After that, they were asked to complete an instances task while controlling the avatar that they created to move in the preset environment in which there was a mirror to let the participants see their faces and also geometric shapes with which they completed the creative task. The result showed that, rather than a non-self-similar avatar, using a self-similar avatar leads to a higher level of originality of ideas which were generated in the task with similarity identification and positive affect as positive mediators. 

Beyond self-similar avatars, using creative avatars also enhances creative performance by inducing the Proteus effect (Guegan et al. 2016). For engineering students, using an inventor avatar (e.g., a scientist in a white lab coat) increases significantly fluency, originality, and self-assessed fluency of the creativity in the group brainstorming session compared to using a non-inventor avatar. Moreover, the Proteus effect induced by inventive avatars still existed in the following face-to-face brainstorming session. 

Another experiment that aimed at examining the creative Proteus effect compared creative ideation under three conditions (i.e., creative, non-creative, and control group), in which the control avatar was represented by an avatar with a similar face and the same clothing as the participant (De Rooij et al. 2017). The results showed that there was no significant difference between the creative and control groups in creative performance. An interpretation of this result from the experimenter was that the avatar in the creative and control group did not help enhance creativity, but the avatar in the non-creative group decreased creativity. However, considering the results that self-similarity positively influences creative performance, the self-similar avatar in similar clothing in the control group might enhance the effect of self-similarity and lead to a higher level of creativity. In other words, an intensively self-similar avatar (i.e., similar face and similar clothing) might have the same boosting effect on creativity as the self-similar creative avatar (i.e., similar face and creative clothing), whose boosting effect has been shown in other experiments (Guegan et al. 2016; Buisine and Guegan 2020).

Beyond the Proteus effect at the individual level, Guegan, Segonds and their colleagues (Guegan et al. 2017a) also conducted an experiment to examine the Social Identity Cues (SICs) theory in the domain of creativity at the collective level. In the experiment, participants were asked or not to wear a costume that represented their identity as a Gadzart (i.e., a student or an alumni from the engineering school ENSAM) and to engage in a group brainstorming session in both physical and VEs. The group wearing the costumes produced more ideas than the group wearing regular clothes because the costume provided social identity cues which allowed participants to believe that they belonged to the group, and put effort into contributing to the group. As the VE provides an anonymous and physically isolated environment that enhances the de-identification of individual and social identification, the effect of social identity cues is stronger in the VE than in the physical environment (Guegan et al. 2017b). In addition, to compare the Proteus effect and the effect of Social Identity Cues, the researcher designed a between-subjects experiment in which four conditions were proposed (i.e., with/without the Proteus effect × with/without social identity cues) (Buisine and Guegan 2020). The results showed that the Proteus effect impacted creative performance only when the social identity cues did not exist. The effect of Social Identity Cues was more powerful than the Proteus effect in terms of creativity enhancement. This might be explained by group homogeneity. Group members are more easily seen as similar to each other than those in another group (Brown 2000). In this case, the creative self-identity cues provided by the Proteus avatar might be less noticeable than the social identity cues provided by the in-group avatar, which results in non-significance of the Proteus effect when the Social Identity Cues effect appears.

**Table 5:** The articles studying different effects induced by avatars in VEs.

Articles	Type of Avatars	Participants	Condition	Results in Terms of Creative Performance
(Marinussen and de Rooij 2019)	Self-similar avatar	57 (M_age_ = 22.02, SD = 4.58, 33F)	Self-similar avatar/non-self-similar avatar	A self-similar avatar positively influenced the **originality** of the ideas rather than a non-self-similar avatar
(De Rooij et al. 2017)	Self-similar avatar; Proteus avatar	61 (M_age_= 23.62, SD = 3.54, 49F)	Creative avatar/non-creative avatar/Control avatar (i.e., self-similar avatar)	Non-creative avatar decreased creativity No significant difference between creative avatar and control avatar
(Guegan et al. 2016)	Proteus avatar	54 (M_age_ = 23.4, SD = 2.7, 9F)	Face-to-face (control condition)/virtual inventive avatar/virtual non-inventive avatar	Participants in the inventor condition produced significantly more (i.e., **fluency**) and unique ideas (i.e., **uniqueness**) than participants in other two conditions
(Guegan et al. 2017a)	Avatar with Social Identity Cues (SICs)	72 (M_age_ = 22.7, SD = 1.9, 15F)	2 (SICs: without/with) × 2 (Setting: face-to-face/virtual)	The group with SIC had a significantly higher **fluency** and **uniqueness** in creativity than the group without SICs No significant difference was found between two settings
(Buisine and Guegan 2020)	Proteus avatar; Avatar with SICs	72 (M_age_ = 23.6, SD = 2.99, 6F)	2 (SICs: without/with) × 2 (Avatar: creative/non-creative)	**Fluency** and **uniqueness** of ideas were higher with creative avatars than with non-creative avatars; **fluency** and **uniqueness** were not affected by SICs The interaction between the Proteus effect and SICs was significant The Proteus effect on **fluency** and **uniqueness** was significant without SICs, whereas the effect of SICs on **fluency** and **uniqueness** was significant for creative avatars

Note: F refers to Female.

#### 4.3.3. The Role of Embodiment in Creative Activities

Embodiment plays a critical role in creative production. Another study showed also a strong power of embodiment on self-evaluation of creativity (Gerry 2017). In this experiment, participants were asked to follow the pre-recorded movement of a painter shown in the headset, and “draw” on an actual canvas. People reported a high level of sense of agency in the post-test session. They reported also an illusion that they followed strictly the movements of the painter and created an artwork, which made them disappointed when finding their actual work was different from the painter’s one. However, another study implied that embodiment was a negative predictor for creativity. De Rooij et al. (2017) found that, with an avatar with the same face and same clothing as the user, participants’ creative performance decreased as the perceived embodiment increased. However, the avatar models in this experiment were made with an instant body scanner which failed to provide high quality data and led to horrible faces on avatars. This might have induced participants’ negative emotions and decreased the Proteus effect. In any case, the strong relation between embodiment and creativity is confirmed, and its orientation should be examined with more experiments using controlled conditions.

An experiment examining the effect of the metaphor “breaking the rules” in a VE used a fNIRS device to record the change in the level of blood oxygen in the brain during the creative process (Wang et al. 2019). The right temporal-parietal junction (r-TPJ) was deactivated under the “break-wall” condition, which indicated that people felt a higher level of embodiment when breaking the wall. Research using other physiological measurements (e.g., fMRI, MRI) has also shown a significant relationship between the deactivation of r-TPJ and better creative performance (Berkowitz and Ansari 2010; Fink et al. 2012). This empirical evidence implies that a higher level of embodiment impacts positively creative performance.

### 4.4. Digital Contextual-Representations: Various Ways to Boost Creativity

#### 4.4.1. VE as a Tool to Influence Cognitive Activities

Simulations of real environments, which involve specific brain regions (e.g., cerebellum) and cognitive processes (e.g., directed attention) in VR systems also induce the same effects as in reality (cf. Table 6). Visual natural stimuli in a virtual setting have a similar impact in real life (Atchley et al. 2012) on creativity. Being immersed in a natural environment induced better creative performance than an urban environment under both 2D (i.e., tablet) and 3D (i.e., headset) settings (Palanica et al. 2019). In addition, exploring biophilic (i.e., indoor environments with nature elements) VEs significantly reduced stress and increased creative performance (Yin 2021), and enclosed biophilic spaces had a stronger impact on creativity than open biophilic spaces.

Dynamic settings as well play an important role in a creativity-boosting VE. Staying in a dynamic VE strengthens the visual perception of body movement and enhances creativity (Fleury et al. 2020). In this study, participants were asked to finish an alternative uses task in either a stationary virtual car or a moving virtual car in which they could see the external lights passing by. The participants in the moving virtual car performed better in the creative task. It should be noted that participants did not move during the whole experimental process, which indicated that visual simulated movement was sufficient to enhance creativity.

**Table 6:** The articles studying the effects of VEs on creative performance by influencing brain functions.

Article	Contextual Effect	Participants	Condition	Results in Terms of Creative Performance
(Palanica et al. 2019) (Study 1)	Nature	84 (M_age_ = 33.6, SD = 7.4, 41F)	Environment (nature/urban) × Medium(mobile tablet/VR headset)	The scores of **fluency**, **flexibility**, **originality** were higher in the nature environment than in the urban environment No difference on the score of **elaboration** was found between two environmental conditions No significant difference was found between two medium conditions
(Yin 2021) (Chapter 3)	Nature	30 (M_age_ = 26.3, SD = 5.1, 22F)	Environment (biophilic (a. Natural elements/b. Natual analogues/c. Combination of a. and b.)/non-biophilic) × workspace types (open/enclosed)	The **score of creative task** was higher in “natural elements” and “combination” conditions than in other environmental conditions The effect of biophilic interventions was significant in enclosed spaces, whereas the effect was not significant in open spaces
(Fleury et al. 2020)	Visual dynamics	32 (M_age_ = 22.1, SD = 2.98, 26F)	Visual movement (stationary/moving)	The **flexibility** of ideas was higher in the motion condition than in the stationary condition

#### 4.4.2. The Contextual-Representations Provide Cues to Induce the Priming Effect and Embodied Metaphor Effect

The contextual-representations in VEs can also enhance creative performance by inducing priming effects (cf. Table 7). Guegan et al. (2017b) designed a creative VE with plants, paintings, books and other elements that people usually associated with creativity. They compared the creative performance of students in two virtual conditions (i.e., virtual creative workspace, virtual meeting room), and a real meeting room. The results suggested that the creative VE had a significant positive impact on originality and elaboration (i.e., the average number of ideas generated for each category). Similarly, an experiment conducted by Bhagwatwar et al. (2018) showed that even if the creative priming element was not target-related (e.g., 3D objects about tourist attraction for a tourism topic), the priming effect still had a positive influence on creativity compared to a generic environment without any priming object. Similarly, Minas et al. (2016) found that, creative performance increased significantly in an open VE (i.e., an outdoor environment without walls), as the concept of “openness” was primed to participants’ mindset when they completed the creative task. 

People exposed to an environment with topic-specific priming objects tend to produce ideas related to those topics. For example, Bhagwatwar et al. (2018) compared the effect of topic-specific and generic VEs on creativity in their first experiment. The researchers prepared two VEs, one with pollution-related (e.g., trash can, windmill) and the other with tourism-related (e.g., theme park, tourist attractions) 3D objects. Participants were then asked to follow brainstorming sessions with the topics “reduce air, water, and land pollution” and “increase tourism in a state”, respectively. Participants generated significantly more topic-related ideas and fewer topic-unrelated ideas in the environments with topic-specific priming objects than in the generic VE. The same effect was found in the first experiment of Nelson and Guegan (2019), in which participants generated more ideas under the forest and underwater categories in the forest and underwater VEs, respectively, regardless of the fact that they were given the same topic for the divergent thinking task. Moreover, in the second experiment, the relationship between details of creative outcomes and the details of primed concepts were analyzed. Two abyss environments, with or without creatures were used, and participants completed the “alien creatures” task in these environments. The results showed that participants in the abyss environment with creatures drew scarier creatures than those in the abyss environment without creatures. This result emphasized again the power of the activation of topic-specific concepts by primed objects.

**Table 7:** The articles studying effects of the contextual cues on creative performance in VEs.

Article	Contextual Effect	Participant	Condition	Results in Terms of Creative Performance
(Guegan et al. 2017b)	Priming	135 (M_age_ = 21.24, SD = 4.11, 107F)	Real control environment (meeting room)/Virtual control environment (meeting room)/creativity-conducive environment (virtual office with the contextual cues most frequently associated with a creativity-inducing environment)	**Originality** and **elaboration** were significantly higher in creativity-conducive environment (CCE) than in virtual control environment (VCE) and real control environment (RCE) **Fluency** was marginally higher in the CCE condition than the other two conditions No significant difference among three conditions was found for **flexibility** and subjective creative perception
(Bhagwatwar et al. 2018) (Study 1)	Priming	168 (M_age_ = 19.8, 70F)	generic VE (i.e., nonspecific conference hall) /topic-specific VE (i.e., conference hall with a trash can, windmill etc. for a pollution topic, and with a theme park, popular tourist attractions etc. for a tourism topic)	The **uniqueness**, **breadth**, and **depth** of ideas were significantly greater in topic-specific VE than generic VE The quality (i.e., **novelty** and **effectiveness**) was higher in topic-specific VE than generic VE The ideas were more target-concept-related and less target-concept-unrelated in topic-specific VE than generic VE
(Bhagwatwar et al. 2018) (Study 2)	Priming	80 (M_age_ = 19.1, 30F)	generic VE (i.e., nonspecific conference hall) /enriched VE (i.e., conference hall with creativity-enhancing and not topic-related 3D objects)	The **uniqueness** and **breadth** of ideas were significantly greater in enriched VE than generic VE The quality (i.e., novelty and effectiveness) was higher in enriched VE than generic VE The ideas were more target-concept-related and less target-concept-unrelated in enriched VE than generic VE
(Nelson and Guegan 2019) (Study 1)	Priming	50 (M_age_ = 22.9, SD = 5.2, 34F)	Forest (a lush green forest surrounded by mountains)/underwater (underwater environment featuring a coral reef)	No significant difference between two conditions in **fluency** and **self-evaluation** of creativity Participants produced more ideas in the underwater category than in the forest category Participants produced more forest-related ideas in the forest condition, and more underwater-related ideas in the underwater condition
(Nelson and Guegan 2019) (Study 2)	Priming	100 (M_age_ = 23.2, SD = 4.89, 49F)	Context (coral reef/abyss) × Creatures (absent/present)	Participants in the environments without creatures produced more drawings than those in the environments with creatures Participants tended to include teeth or tentacles more in abyss environment than coral reef environment Participants in the abyss environment without creatures tended to include tentacles in the drawings Participants in the abyss environment with creatures tended to include teeth in the drawings Participants in environments without creatures generated more original ideas than others in environment with creatures Participants generated more ideas in the abyss condition without creatures than with creatures, whereas no difference was found in coral reef environments Participants in the abyss environment produced more scarier drawings in the abyss environment than in the coral reef environment Participants in the abyss environment with creatures produced scarier drawings than those in the abyss environment without creatures, whereas no difference was found in coral reef environments
(Minas et al. 2016)	Priming	140 (age M = 19.8, SD = 1.78, 63F)	Open space without wall/closed space with wall	The **originality**, **feasibility**, and **relevance** was higher in the open space than closed space
(Wang et al. 2019)	Embodied metaphor	90 (age M = 21.55, SD = 1.98, 67F)	Break-wall/auto-wall/no-wall	**Originality**, **fluency**, and **flexibility** were higher in break-wall condition than other two conditions

Note: F refers to Female.

In the study by Wang et al. (2019), participants were asked to walk in a virtual corridor, with or without walls as obstacles, on their way when finishing the divergent thinking task. The participants in the “break-wall” group were required to break the wall to present the metaphor “breaking the rules”, the wall disappeared when participants were nearby in the “auto-wall” condition, and the wall did not exist in the “no-wall” condition. The results showed that the originality of ideas increased over time in all conditions. However, the participants who had the “break the rules” metaphor created new ideas more rapidly, which indicated that the metaphorical rule breaking induced better creative productions (Wang et al. 2019).

#### 4.4.3. The Role of Presence

Men and colleagues conducted a series of experiments (Men and Bryan-Kinns 2018, 2019; Men and Bryan-Kinns 2019) with the self-developed music-composing software “LeMo” to explore the relationship among working space, sense of presence, and creativity. LeMo is software with which participants work together to make music clips. The difference among experimental conditions in three experiments were the division of private and public spaces in LeMo. In the initial experiment (Men and Bryan-Kinns 2018), only one fixed shared space was provided for people to communicate and share ideas. Later, researchers provided three types of combinations of private and public virtual space in LeMo: (1) one shared space and no private space, so that people stayed together during the whole collaboration process; (2) one shared space and two invisible spaces where people could not see or hear each other (the absence of co-presence); and (3) one shared space and two visible spaces where people can hear and see each other (the existence of co-presence). Interviews related to the affordance of LeMo and the measure of presence using the Igroup presence questionnaire were conducted with participants after their collaborations in LeMo. The responses showed that the private space inhibited more or less collective creativity. Meanwhile, individual creativity was supported in such private areas (Men and Bryan-Kinns 2019). As a follow-up experiment, more division types were introduced in LeMo (Men et al. 2019). Except for the conventional (1) only-public space and (2) public space with fixed personal space, two more division types were designed: (3) the public space with augmented attenuation personal space which can be considered as a simulation of real-life scenarios where the volume of audio dropped faster over distance, and (4) public space with moveable personal space, which has similar features to the fixed personal space, but matches the real-time positions of users. The public space with fluid personal space maintained self-presence and co-presence and encouraged individual and collective creativity simultaneously.

## 5. Implications and Limitations

In this paper, we discussed how different types of digital users’ representations and digital contextual cues influence creative performance in VEs. We provided some examples to show the role of embodiment and presence in enhancing creativity in VEs. After analyzing the effects induced with immersion and digital representations, we can conclude that (1) Immersion is a technical feature that helps boost creative performance, but is restricted under some conditions (e.g., no human-VE interaction). (2) According to research purposes, users can be represented in various ways, such as virtual controllers, avatars that match users’ gender; avatars with individual or social identity cues; or even absent. (3) At the individual level, self-similar avatars and creative avatars can both enhance creative performance, whereas avatars with SICs can help enhance collective creativity. (4) Contextual-representations support creative activities in VEs in various ways such as inducing brain activities that are related to the creative process or providing creative-related mindsets. (5) Embodiment has also a positive impact on creative activities in VEs. (6) Presence influences users’ attitude towards collective creativity and the quality of collaboration in VEs. 

The current paper provides a map categorizing the most common creative effects related to digital representations in recent years and the recent empirical studies that compare the effectiveness of two VEs. It helps researchers to distinguish different creative effects induced by digital representations, but also to find a gap between future research topics and existing empirical studies. From the practical perspective, the article provides evidence about how VR technologies impact creative outcomes, which is useful for VR developers, teachers, and managers in industries who want to apply VR technologies to classrooms or professional scenarios and customize VEs to match various needs. 

More importantly, the results indicate that the VE might be able to provide a new, ecological platform to help find solutions to the challenges in the Anthropocene. First, as the IVE supports a high level of embodiment, presence and flexibility to customize both the virtual context and avatar to match different purposes, which encourages the interaction and the collaboration among users, it might be used as a virtual classroom or virtual workplace. The COVID crisis changed a lot the ways that people study and work, and showed that permanent remote work or study were feasible under some conditions, and that some resources (e.g., electricity, buildings) used to support work or education could be saved. However, the conventional multi-user collaboration platforms, such as Zoom or Microsoft Teams do not support a wide range of customization, such as changing the virtual working environment for all the team members. VEs, in contrast, can provide virtual platforms that keep the advantages of conventional virtual platforms, but reduce the restrictions of virtual collaboration, and improve efficiency. Second, as creativity could be enhanced in some VEs because of the common features of VEs (e.g., immersion, anonymity) compared to the physical world, and also user and contextual representations which are relatively stable in the physical world, VEs might be superior to the physical world as a space where people engage in innovative production. Therefore, VEs would be an excellent environment for experts who work on solutions to challenges in the Anthropocene to produce more innovative and feasible solutions to deal with the threats we are facing in the physical world.

However, the analysis of selected studies is limited. As immersive VR technology is still developing and is not widely used yet, studies comparing two or more VEs in terms of their creative-supporting effects are still lacking (in our case, only three articles comparing two environments were collected). More importantly, the inconsistency among results indicates that further empirical studies are needed to examine the mechanism of immersion in boosting creativity.

Moreover, the effects of embodiment and presence are still overlooked by researchers when designing experiments. First, in many studies, embodiment, and presence played a role as control variables which showed the similarity of VEs at the same level of immersion (e.g., Guegan et al. 2017b), or dependent variables which showed the level of immersion for the VEs (e.g., Gerry 2017). Indeed, the different levels of embodiment and presence result from the difference in immersion. When researchers examine the creative effect in a single type of VE (i.e., either a nIVE or IVE), the influences of embodiment and presence are not significant. Further research that compares two VEs should consider the influences of these two psychological states under different levels of immersion, positive or negative, on creativity to avoid a biased result. Second, many studies mixed up the definition of “embodiment” and “presence”, which made the results unclear. Moreover, the measurement of embodiment and presence are often presented with one or two questions instead of reliable scales. For example, an experiment studying the effect of creative metaphor (Wang et al. 2019) used the question “How much did you feel immersed in the scene?” to evaluate the sense of embodiment. However, the question evaluated the sense of spatial presence as the question was related to the immersion in the scene instead of the virtual avatar. Therefore, future studies should distinguish the two psychological states and find suitable scales as measurement tools. Third, the relationship among embodiment, presence, and creative performance needs to be determined with more empirical evidence. Though we read several conclusions that embodiment, presence, and creative performance increased significantly in an immersive environment, only a few studies conducted correlational analyses between the two psychological states and creativity. Furthermore, the results of correlational analyses between presence and behavior in the VE were inconsistent. Some studies found no relationship between presence and performance (Slater et al. 1996), whereas others found a significant impact of presence on behavior (Yassin et al. 2021). As creative performance relies strongly on contextual cues, we hypothesize that presence would play an important role in fostering creativity in the VE.

According to the findings and limitations of the selected papers, we suggest researchers should consider the following orientations for further studies related to VEs and creativity: (1) examine the effect of embodiment and presence in boosting creativity in IVE and nIVE with accurate definitions and measurements for the two psychological states, and (2) examine the effects of digital representations with larger sample sizes to provide more empirical data about the effectiveness of creative effects of digital representations as some effects are supported by only one or two empirical studies (e.g., the effect of self-similar avatar, visual dynamics, or embodied metaphor).

There are also some limitations of this article. First, this review focused on analyzing the boosting effect of virtual avatars and contextual cues on creative performance. However, creativity in the VE is also influenced by communication modalities (Forens et al. 2015), the combination of different platforms in creative activities (Agnès et al. 2022), and user experience (Hong et al. 2016; Fröhlich et al. 2018). These factors are also worth discussing, as they influence creative performance in a positive or negative way. In addition, we focused on the lab experiments in which the unrelated variables were strictly controlled to have a more precise analysis. However, we failed to include the results from workshops or cases studies (e.g., Bujdosó et al. 2017), which simulate more practical and real scenarios of creative activities in VR systems. 

## 6. Conclusions

In summary, several empirical studies have provided a basis for the application of digital representations (i.e., avatars and contextual cues) to boost creativity in VEs. The roles and potential effects of one’s perceptions (i.e., embodiment and presence) in the VEs were also discussed. The results indicated creativity can be influenced positively by high levels of immersion, embodiment, and presence. Moreover, digital representations boost creativity in VEs in diverse ways. The next step is to examine if “immersion” strengthens such effects in an IVE. Further, if so, the roles of embodiment and presence in the creativity enhancement process, and the intensity of their effects on virtual creative activities can be studied. This will facilitate the construction of future virtual environments, which will ultimately co-exist with physical ones in the Anthropocene era.

## Figures and Tables

**Table 2:** The types of VEs (non-immersive/immersive) used in articles between 2016 and 2021.

Publication Year	Type of VEs Used in Studies		
	nIVE	IVE	nIVE & IVE
2016	(Guegan et al. 2016; Hong et al. 2016; Minas et al. 2016)	/	/
2017	(Guegan et al. 2017a, 2017b)	(De Rooij et al. 2017); (Gerry 2017)	/
2018	(Bhagwatwar et al. 2018)	(Men and Bryan-Kinns 2018)	(Parmar 2018)
2019	/	(Marinussen and de Rooij 2019; Men and Bryan-Kinns 2019; Men et al. 2019) (Nelson and Guegan 2019); (Wang et al. 2019)	(Palanica et al. 2019)
2020	(Bourgeois-Bougrine et al. 2020); (Buisine and Guegan 2020)	(Fleury et al. 2020)	(Obeid and Demirkan 2020)
2021	/	(Yin 2021)	/

Note: nIVE = non-Immersive Virtual Environment, IVE = Immersive Virtual Environment, nIVE & IVE = comparison between non-Immersive and Immersive Virtual Environment, “/” means not applicable.

**Table 3:** The methods used in studies examining individual creativity.

Article	Device	Participants	Virtual Platform	Creative Task
(De Rooij et al. 2017)	Oculus Rift DK2	61 (age M = 23.62, SD = 3.54, 49F)	Self-developed scene	The instances task
(Fleury et al. 2020)	HTC Vive	32 (age M = 22.1, SD = 2.98, 26F)	Self-developed scene	(a) Alternative Uses Test (b) Remote Association Test
(Gerry 2017)	Oculus Rift DK2	32 (age M = 27, SD = 7.2, 15F)	Self-developed scene	Design task: move along with the painter in the VR headset and follow her movements as much as possible to “create” a work of art
(Guegan et al. 2017b)	/	135 (age M = 21.24, SD = 4.11, 107F)	Second Life	Cardboard boxes task
(Marinussen and de Rooij 2019)	HTC Vive	57 (age M = 22.02, SD = 4.58, 33F)	Self-developed scene	The instances task
(Nelson and Guegan 2019)	Oculus Rift DK2	Experiment 1: 50 (age M = 22.9, SD = 5.2, 34F) Experiment 2: 100 (age M = 23.2, SD = 4.89, 49F)	Self-developed scene	(a) Variant of Cardboard boxes task (b) The “alien creatures” task
(Obeid and Demirkan 2020)	Oculus Rift DK2; iPad mini A1432	42 (29F)	Gravity Sketch	Design task: creative 3D composition that considers meaningful combinations, either following a certain sequence, pattern or order of the geometric forms using the fundamental basic design principles
(Palanica et al. 2019)	Oculus Rift; 12.9” iPad Pro	E1: 84 (age M = 33.6, SD = 7.4, 41F) E2: 97	Videos	Alternative Uses Test
(Parmar 2018)	Oculus Rift; Kinect motion sensor	90 (59F)	VEnvI	Design task: choreography
(Wang et al. 2019)	NA	90 (age M = 21.55, SD = 1.98, 67F)	Self-developed scene	Alternative Uses Test
(Yin 2021)	HTC Vive	30	Self-developed scene	Alternative Uses Test

Note: NA means “not specified”; “/” means not applicable; “F” means female.

**Table 4:** The methods used in studies examining collective creativity.

Article	Number of Groups	Number of Members in Each Group	Virtual Platform	Creative Task
(Bhagwatwar et al. 2018)	Study 1: 42 Study 2: 20	4	Open Wonderland	Brainstorming: (a) increase tourism in a state; (b) reduce air, water, and land pollution
(Bourgeois-Bougrine et al. 2020)	20	3	Second Life	Brainstorming: improve the daily mobility in the Paris region
(Buisine and Guegan 2020)	24	3 (including 1 or 0 female)	Second Life	Brainstorming: (a) imagine a crazy solution for travelling on snow, sand or Water (b) imagine a silent flying public transportation for the future
(Guegan et al. 2016)	18	3	Second Life	Brainstorming: (a) imagine a crazy solution for traveling on snow, sand, or water; (b) imagine a silent flying public transportation for the future
(Guegan et al. 2017a)	24	3	Second Life	Brainstorming: imagine new transportations means
(Hong et al. 2016)	22	2	Second Life; Group Board	Design task: (a) two bus stops; (b) two street exhibition booths
(Men and Bryan-Kinns 2018)	16	2	Self-developed software	Design task: music composition
(Men and Bryan-Kinns 2019)	21	2	Self-developed software	Design task: music composition
(Men et al. 2019)	26	2	Self-developed software	Design task: music composition
(Minas et al. 2016)	35	4	Open Wonderland	Brainstorming: (a) increase tourism within the state; (b) reduce pollution

## Data Availability

No data were created for this article.

## Notes

1 Although the term “immersion” was used by some authors to describe a psychological experience that participants reported in a VE (e.g., Zhang et al. 2017), in this article, we use only its technical aspect to avoid the ambiguity between technological and psychological factors of “immersion” and their effects on users.

2 Notice that the word “embodied” is distinct from the concept “embodiment” in this article. The word “embodied” here is related to the term “embodied cognition” which means that cognition is influenced by body action (Shapiro and Stolz 2019).
